# Retraction: Stereochemical Insignificance Discovered in *Acinetobacter baumannii* Quorum Sensing

**DOI:** 10.1371/annotation/8f2ddf91-3499-4627-9a91-449b78465f9d

**Published:** 2013-04-24

**Authors:** Amanda L. Garner, Sook Kyung Kim, Jie Zhu, Anjali Kumari Struss, Richard Watkins, Brent D. Feske, Gunnar F. Kaufmann, Kim D. Janda

Retraction notice

The editors retract this publication following the recommendation issued by Central South University as a result of an investigation over concerns about duplicate publication of some of the data reported in the article. 

Following the publication of the article, it was brought to the editors’ attention that that the data reported in the article are very similar to that reported the authors' related publication below:

miR-338-3p Is Down-Regulated by Hepatitis B Virus X and Inhibits Cell Proliferation by Targeting the 3'-UTR Region of CyclinD1

Fu X, Tan D, Hou Z, Hu Z, Liu G.

Int J Mol Sci. 2012;13(7):8514-39. Epub 2012 Jul 9.

Upon evaluation of the two articles, the editors noted the following overlap in the data reported:

- Figures 1A and 1B in the PLOS ONE article correspond to Figures 3A and 3B in the Int J Mol Sci article

- The data in Figure 2 in the PLOS ONE article correspond to in Figures 3C and 3D in the Int J Mol Sci article

- The FACS data in Figure 3 in the PLOS ONE article are similar to those in Figure 4 in the Int J Mol Sci article

- Figure 4 "Mimics LO2/HBx-d382" in the PLOS ONE article corresponds to Figure 5 "Mimics LO2/HBx" in the Int J Mol Sci article

- Figure 6C in the PLOS ONE article corresponds to Figure 6b in the Int J Mol Sci article

- Figure S3 in the PLOS ONE article overlaps with the data in Figure 7 in the Int J Mol Sci article

- Figure S2 in the PLOS ONE article overlaps with the data in Figure 4 in the Int J Mol Sci article

The publication in International Journal of Molecular Sciences was not cited in the PLOS ONE article. The editors were also concerned about a selective representation of data, given that several Western Blots included in the International Journal of Molecular Sciences article are missing lanes that are displayed in the PLOS ONE article.

The editors contacted the authors for their comments and they acknowledged that some of the data overlap between the two publications. The editors referred this matter to Central South University to request an investigation into the concerns identified about the article. The representatives of Central South University have completed their investigation and they have requested the retraction of the PLOS ONE article.

